# Synthesis, analgesic and anti-inflammatory activities of new methyl-imidazolyl-1,3,4-oxadiazoles and 1,2,4-triazoles

**DOI:** 10.1186/2008-2231-22-22

**Published:** 2014-01-22

**Authors:** Ali Almasirad, Zahra Mousavi, Mohammad Tajik, Mohammad Javad Assarzadeh, Abbas Shafiee

**Affiliations:** 1 Department of Medicinal Chemistry, Pharmaceutical Sciences Branch, Islamic Azad University, Tehran, Iran; 2 Department of Toxicology and Pharmacology, Pharmaceutical Sciences Branch, Islamic Azad University, Tehran, Iran; 3 Department of Medicinal Chemistry, Faculty of Pharmacy and Pharmaceutical Sciences Research Center, Tehran University of Medical Sciences, Tehran, Iran

**Keywords:** Analgesic, Anti-inflammatory, Non ulcerogenic, Imidazole, Oxadiazole, Triazole

## Abstract

**Background:**

Long-term clinical employment of nonsteroidal anti-inflammatory drugs (NSAIDs) is associated with significant side effects including gastrointestinal (GI) lesions and kidney toxicity. In this paper we designed and synthesized new imidazolyl-1,3,4-oxadiazoles and 1,2,4-triazoles by molecular hybridization of previously described anti-inflammatory compounds in the hope of obtaining new safer analgesic and anti-inflammatory agents.

**Methods:**

The target structures were synthesized by preparation of 5-methyl-1H-imidazole-4-carboxylic acid ethyl ester 5. The reaction of hydrazine hydrate with this ester afforded the 5-methyl-1H-imidazole-4-carboxylic acid hydrazide 6 which was converted to target compounds 7-15 according to the known procedures. *In silico* toxicity risk assessment and drug likeness predictions were done, in order to consider the privileges of the synthesized structures as drug candidates.

**Results and discussion:**

The analgesic and anti-inflammatory profile of the synthesized compounds were evaluated by writhing and carrageenan induced rat paw edema tests respectively. Compounds 8, 9 and 11-13 and 15 were active analgesic agents and compounds 8, 9 and 11-13 showed significant anti-inflammatory response in comparison with control. Compounds 11 and 13 were screened for their ulcerogenic activities and none of them showed significant ulcerogenic activity. The active Compounds 11 and 12 showed the highest drug likeness and drug score.

**Conclusions:**

The analgesic and anti-inflammatory activities of title compounds were comparable to that of standard drug indomethacin with a safer profile of activity. The results revealed that both of oxadiazole and triazole scaffolds can be determined as pharmacophores. The *in silico* predictions and pharmacological evaluations showed that compounds 11 and 12 can be chosen as lead for further investigations.

## Background

**Figure 1:**
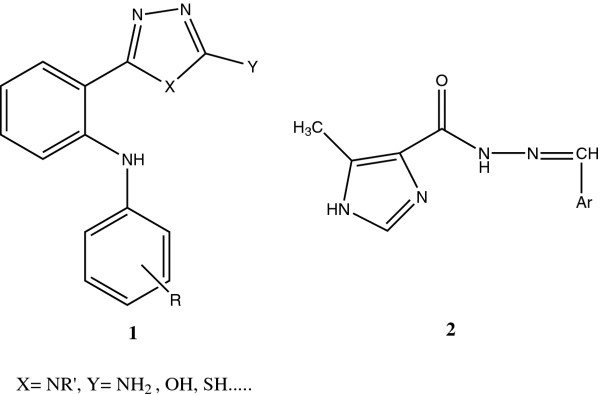
Chemical structures of compounds 1 and 2 as dual COX/5-LO inhibitors and anti-inflammatory agents.

Cyclooxygenase (COX) and 5-Lipoxygenase (5-LO) are two key enzymes that play important roles in the metabolism of arachidonic acid (AA) to pro-inflammatory prostaglandins (PGs) and leukotrienes (LTs) respectively [1]. It has been pointed out that inhibiting COX enzyme could decrease cytoprotective PGs and increase the conversion of AA to LTs by 5-LO enzyme which are implicated in the ulceration induced by nonsteroidal anti-inflammatory drugs (NSAIDs) [2,3]. Conversion of AA to Leukotrien B_4_ (LTB_4_) leads to hyperalgesic response and related with rheumatoid arthritis and inflammatory bowel disease. However, dual inhibition of COX and 5-LO would provide an anti-inflammatory agent with better efficacy and fewer side effects [4-6]. During recent years, various structural families such as di-tert-butylphenols, thiophene and pyrazoline derivatives and modified NSAIDs have been investigated as dual COX/5-LO inhibitors [7-9]. Amongst them several 1,3,4-oxadiazole, 1,3,4-triazole and hydrazone derivatives, compounds 1 and 2 were dual COX, 5-LO inhibitors and anti-inflammatory agents (Figure 1) [7-12]. As a part of our ongoing research program to find novel anti-inflammatory and analgesic compounds, herein,we describe the synthesis of new imidazolyl-oxadiazole and triazole derivatives which were designed by molecular hybridization of previously described compounds, 1 and 2, in order to evaluate the plausible pharmacophoric contribution of the both imidazole and oxadiazole or triazole moieties in the pharmacological activities. *In silico* toxicity risk assessment and drug likeness predictions were done, in order to consider the privileges of the synthesized structures as drug candidates.

## Methods

### Chemistry

Chemicals were purchased from Merck Chemical Company (Tehran, Iran) and carrageenan was prepared from (Sigma-Aldrich, Dorset, UK). Melting points were taken on an electrothermal IA 9300 capillary melting-point apparatus (Ontario, Canada) and are uncorrected. ^1^H-NMR spectra were obtained using a Bruker FT-400 spectrometer (Bruker, Rheinstetten, Germany). Tetramethyl silane was used as an internal standard. Mass spectra were obtained using a 5973 Network Mass Selective Detector at 70 eV (Agilent Technology). The FT-IR spectra were obtained using a Shimadzu FT-IR 8400S spectrographs (KBr disks).

### 5-Methyl-1H-Imidazole-4-carboxylic acid hydrazide (6)

To a stirring solution of ester 5 (10 g, 71.5 mmol) in 125 ml ethanol, at room temperature hydrazine hydrate (50 ml, 1000 mmol) was added and refluxed for 48 h. The resulting solution was concentrated, cooled and the precipitate was filtered, washed with dichloromethane and crystallized in ethanol to give 6.73 g (74%) of 6, mp 214-217°C. IR (KBr): υ cm^-1^ 3326, 3270 (NH_2_, NH), 1656 (C = O). ^1^H-NMR(DMSO-d6): δ(ppm) 11.95 (bs, 1H,NH), 8.61(bs, 1H, NH), 7.40(s, 1H, CH, imidazole), 4.09(bs, 2H, NH2), 2.51(s, 3H, CH3). MS: m/z (%) 140(M+, 100), 109(78), 54(20).

### 2-Amino-5-(5-methy-1H-imidazol-4-yl)-1,3,4-oxadiazole (7)

To a stirring suspension of hydrazide 6 (500 mg, 3.57 mmol) in dioxane (12 ml), sodium bicarbonate (300 mg) in water (1 ml) was added at room temperature. The mixture was stirred at room temperature for 5 min and cyanogen bromide (386 mg, 3.64 mmol) was added. After 24 h stirring at room temperature the solvent was evaporated and the resulting precipitate was purified by crystallization in methanol to give 400.7 mg(68%) of 7, mp 300-301°C. IR (KBr): υ cm^-1^ 3390, 3349 (NH_2_, NH), 3135 (C-H). ^1^H-NMR (DMSO-d_6_): δ (ppm) 8.21 (bs, 1H, NH), 7.55(s, 1H, CH, imidazole), 6.85(bs, 2H, NH_2_), 2.44(s, 3H, CH_3_). MS: m/z (%) 165(M^+^, 18), 69(58), 45(100).

### 5-(5-Methyl-1H-imidazol-4-yl)-1,3,4-oxadiazole-2(3H)-thione (8)

A mixtue of hyrazide 6 (500 mg, 3.57 mmol), potassium hydroxide (200 mg, 3.57 mmol), carbon disulfide (0.64 ml, 10.7 mmol) and ethanol (30 ml) was heated under reflux for 72 h. The solution was acidified with dilute hydrochloric acid. The resulting precipitate was removed by filtration and purified by crystallization in methanol to give 6.73 g (15%) of **8**, mp 261-262°C. IR (KBr): υ cm^-1^ 3477, 3281 (NH), 1626 (C = N), 1338 (C = S). ^1^H-NMR (DMSO-d_6_): δ (ppm) 13.97 (bs, 1H, NH), 8.20(bs, 1H, NH), 7.71(s, 1H, CH, imidazole), 2.43(s, 3H, CH_3_). MS: m/z (%) 182(M^+^, 100), 122(87), 109(100).

### 5-(5-Methyl-1H-imidazol-4-yl)-1,3,4-oxadiazole-2(3H)-one (9)

To a solution of compound 6 (550 mg, 4 mmol) and triethyl amine (400 mg, 4 mmol) in DMF (50 ml), 1,1′-carbonyldiimidazole (0.84 g, 5.1 mmol) was added in one portion. The reaction mixture was refluxed for one week. The volatiles were removed in vacuo, and the residue was purified by TLC, eluting with ethyl acetate-petroleum ether (1:1) to provide 78.7 mg (11%) of **9**, mp 298-299°C. IR (KBr): υ cm^-1^ 3332(NH), 3206(NH), 1714(C = O). ^1^H-NMR (DMSO-d_6_): δ (ppm) 9.1(bs, 1H, NH), 8.1(bs, 1H,NH), 7.55(s, 1H, CH, imidazole), 2.43(s, 3H, CH_3_). MS: m/z (%) 166(M^+^, 10), 140(18), 109(100).

### 5-(5-Methyl-1H-imidazol-4-yl)-4H-1,2,4-triazole-3-thione (10)

A solution of hydrazide 6 (500 mg, 3.57 mmol), potassium thiocyanate (1 g, 10.71 mmol), concentrated hydrochloric acid (4 ml) and water 50 ml was stirred at room temperature for 24 h. To the resulting suspension, sodium hydroxide 4% (60 ml) was added and stirred for 24 h at 60-70°C.The resulting mixture was neutralized with hydrochloric acid and the resulting precipitate was filtered, washed with acetone and purified by two times crystallization in ethanol to give 219 mg (34%) of **10**, mp 225-227°C. IR (KBr): υ cm^-1^ 3400, 3134 (NH), 3114 (C-H), 1321 (C = S). ^1^H-NMR (DMSO-d_6_): δ (ppm) 13.87(bs, 1H, NH), 12.25(bs, 1H, NH), 9.1(bs, 1H, NH), 7.41(s, 1H, CH, imidazole), 2.31(s, 3H, CH_3_). MS:m/z (%) 181(M^+^, 13), 125(100), 107(90), 80(82), 67(58).

### General procedures for the preparation of 4-substituted-5-(5-methyl-1H-imidazol-4-yl)-4H-1,2,4-triazole-3-thiones (11-15)

A mixture of compound 6 (500 mg, 3.57 mmol), corresponding isothiocyanate derivative (3.6 mmol) and absolute ethanol (5 ml) was stirred for 24 h. The resulting semicarbazide was filtered and washed with ether then added to 3% sodium hydroxide solution (50 ml) and refluxed for 24-48 h. The resulting solution was acidified with hydrochloric acid, the precipitate was filtered, dried and purified by crystallization.

### 4-Methyl-5-(5-methyl-1H-imidazol-4-yl)-4H-1,2,4-triazole-3-thione (11)

Yield 34%; mp 225-227°C (ethanol). IR (KBr): υ cm^1-^ 3376, 3149 (NH), 2526(weak, SH), 1337 (C = S). ^1^H–NMR (DMSO-*d*_*6*_): δ(ppm) 12.12(bs, 1H, NH), 8.25(bs, 1H, NH), 7.72(s, 1H, CH, imidazole), 3.74(s, 3H, CH3), 2.37 (s, 3H, CH_3_). MS: m/z (%) 195(M^+^, 100), 122(25), 108(30).

### 4-phenyl-5-(5-methyl-1H-imidazol-4-yl)-4H-1,2,4-triazole-3-thione (12)

Yield 13%; mp 275-277°C (THF/Water). IR (KBr): νcm^1-^ 3477(NH), 3410(NH), 3118(C-H, aromatic), 1326 (C = S). ^1^H–NMR (DMSO-*d*_*6*_): δ (ppm) 12.39 (bs, 1H, NH), 8.5 (bs, 1H, NH), 7.6-7.2(m, 6H, aromatic and CH, imidazole), 2.26 (s, 1H, CH_3_). MS: m/z (%) 257(*M*^*+*^, 100), 122(25), 108(30).

### 5-(5-Methyl-1H-imidazol-4-yl)-4-(4-methylphenyl)-4H-1,2,4-triazole-3-thione (13)

Yield 24%; mp 268-270°C (Methanol). IR (KBr): υ cm^-1^ 3434(NH), 3210(NH), 3100(C-H,aromatic), 1340 (C = S). ^1^H–NMR (DMSO-*d*_*6*_): δ (ppm) 12.2(bs, 1H, NH), 8.5(bs, 1H, NH), 7.4(s, 1H, CH, imidazole), 7.3(d, J = 7.9Hz, 2H, aromatic), 7.1(d, J = 7.9 Hz, 2H, aromatic), 2.34(s, 1H, CH_3_), 2.25(s, 3H, CH3). MS: m/z (%) 271(M^+^, 20), 257(100), 106(22), 77(30).

### 5-(5-Methyl-1H-imidazol-4-yl)-4-(4-methoxyphenyl)-4H-1,2,4-triazole-3-thione (14)

Yield 18%; mp 140-142°C (Ethylacetate/Acetone). IR (KBr): υ cm^-1^ 3430(NH), 3216(NH), 3144(C-H,aromatic), 1311(C = S). ^1^H–NMR (DMSO-*d*_*6*_): δ (ppm) 12.8(bs, 1H, NH), 8.5(bs, 1H, NH), 7.4(s, 1H, CH, imidazole), 7.2(d, J = 8.4Hz, 2H, aromatic), 6.9(d, J = 8.4Hz, 2H, aromatic), 3.76(s, 3H, OCH_3_), 2.27(s, 3H, CH3). MS: m/z (%) 287(M^+^, 100), 106(22), 77(30).

### 5-(5-Methyl-1H-imidazol-4-yl)-4-(4-fluorophenyl)-4H-1,2,4-triazole-3-thione (15)

Yield 20%; mp 150-152°C (Methanol). IR (KBr): υ cm^-1^ 3467 (NH), 3365 (NH), 3108(C-H, aromatic), 2520(weak, SH), 1321 (C = S). ^1^H-NMR (DMSO-*d*_*6*_): δ (ppm) 12.4 (bs, 1H, NH), 8.5 (bs, 1H, NH), 7.8-7.3 (m, 5H, aromatic and CH, imidazole), 2.25(s, 3H, CH_3_). MS: m/z (%) 275(M^+^, 100), 106(22), 77(30).

### Pharmacology

Male NMRI mice (20-25 g) and Wistar rats (100-150 g) were purchased from the animal breeding laboratories of Pasteur Institute (Karaj, Iran). Each group consisted of six animals. The animals were maintained in colony cages at 25 ± 2°C, relative humidity 45–55%, under a 12 h light-dark cycle; they were fed standard animal feed. All the animals were acclimatized for a week before use and all ethical manners for use of laboratory animals were considered carefully and the protocol was approved by Islamic Azad University of Pharmaceutical Sciences (IAUPS) ethical committee.

### Analgesic activity evaluation

Acetic acid writhing test was performed on mice and indomethacin was used as standard drug [11]. Test compounds and the standard drug were administered intraperitoneally (ip) to the animals at the dose of 50 μmol/kg as suspension in saline and tween 80 (4% w/v). An acetic acid (0.6%, 0.1 ml/10 g) solution was administered ip 30 minutes after administration of compounds. The mean number of writhes for each experimental group and percentage decrease, compared with that of the control group were calculated during 30 minutes.

### Anti-inflammatory activity against carrageenan induced rat paw edema

Effective compounds in analgesic test were screened for their anti-inflammatory activities using carrageenan induced paw edema on wistar male rats [13]. The standard and test groups received indomethacin as standard drug or target compounds, 50 μmol/kg body weight (ip) suspended in 4% tween in saline and the control animals received 4% tween in saline respectively. One hour after administration of drugs each rat received freshly prepared 0.1% w/v aqueous solution of carrageenan in the sub plantar region of right hind paw. The paw thickness was measured from the ventral to the dorsal surfaces using a dial caliper immediately prior to carrageenan injection and then at each hour, up to 5 h after the sub plantar injection. The edema was calculated as the thickness variation between the carrageenan and saline treated paw. Anti-inflammatory activity was expressed as the inhibition percent of the edema when compared with the control group.

### Ulcerogenic activity

The ulcerogenic activity of newly synthesized compounds relative to known ulcerogenic drug, indomethacin was evaluated and scored by method of Cioli *et al.*[14]*.* Wistar rats (160-180 g) were fasted for 24 h before giving a single dose of each of vehicle, standard and test compounds (105 mg/kg in 0.5% v/v CMC suspension, peroral respectively and 17 h later, sacrificed under deep ether anesthesia and stomachs were removed and then examined by means of a magnifying glass to assess, the incidence of redness and spot ulcers. For each stomach, the mucosal damage was evaluated according to the following scoring system: 0.5: redness; 1.0: spot ulcers; 1.5: hemorrhagic streaks; 2.0: ulcers >3 but ≤5; 3.0: ulcers > 5. The mean score of each treated group minus the mean score of control group was regarded as gastric mucosal ulceration score.

### In silico drug-score and toxicity assessments

Currently, there are several approaches to assess the drug-likeness of the drug candidates [15,16]. One such tool is the Osiris Property Explorer (OPE) a web based system which is able to calculate properties such as toxicity risk assessment and prediction of log p, solubility (log s), fragment-based drug-likeness and overall drug-score.

## Results and discussion

### Chemistry

**Scheme 1:**
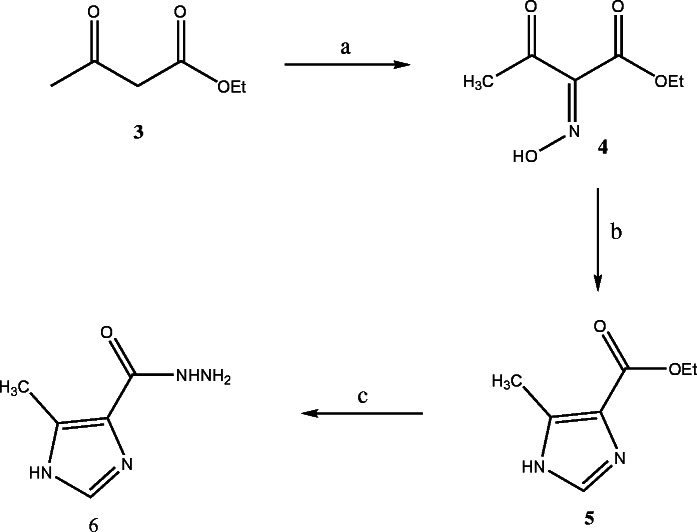
**Synthesis of hydrazide 6. (a)** CH_3_COOH, NaNO_2_, 0°C; **(b)** HCHO, HCl, 0-5°C; NH_3_, 70°C; **(c)** NH_2_NH_2_.H_2_O, EtOH, reflux.

**Scheme 2:**
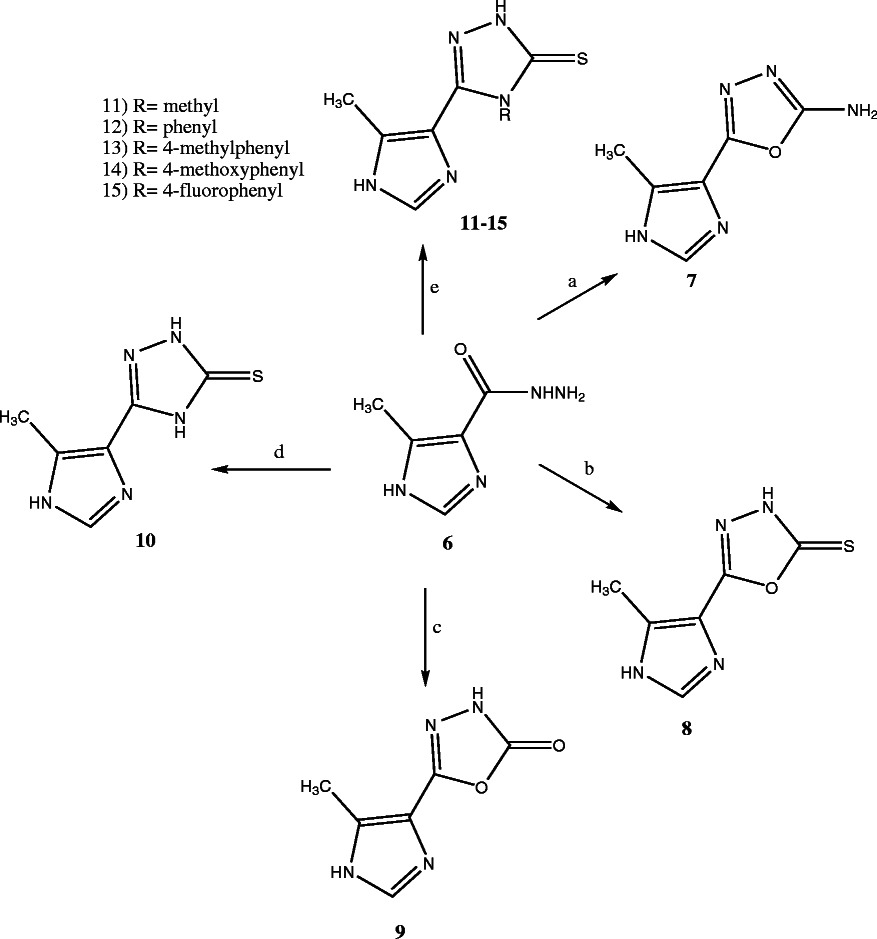
**Synthesis of target derivatives 7–15. (a)** BrCN, Dioxane, rt; **(b)** CS_2_, KOH, EtOH, reflux; **(c)** 1,1′-CDI, triethylamine, DMF, reflux; **(d)** (1) KSCN, HCl, H_2_O, rt; (2) NaOH 3%, 60-70°C; **(e)** (1) Isothiocyanate derivatives, EtOH, rt; (2) NaOH, reflux.

The target compounds were prepared according to the Schemes 1 and 2. The ester 5 was synthesized as previously described method [17]. The key intermediate 6 was prepared from the reaction of hydrazine hydrate with ester 5. Reaction of hydrazide 6 with cyanogens bromide gave 2-amino-1,3,4-oxadiazole 7 [18]. The 1,3,4-oxadiazole-2-thone 8 and the 1,3,4-oxadiazole-2-one 9 were prepared by the reaction of hydrazide 6 with KOH and CS_2_ or 1,1′-carbonyldiimidazole (1,1′-CDI) in the presence of triethylamine respectively [18,19]. The 1,2,4-triazole-3-thione 10 was synthesized via reaction of 6 with potassium thiocyanate and hydrochloric acid followed by cyclization of thiosemicarbazide intermediate with aqueous sodium hydroxide. Reaction of 6 with corresponding isothiocyanates yielded thiosemicarbazides as intermediate which were converted to 4-substituted-1,2,4-triazole-3-thiones **11-15** in aqueous sodium hydroxide [20-22]. The FCH group, Ukraine is the supplier of compound 11 and the synthesis of compound 12 has been previously reported [23].

### Analgesic activity

The results of writhing test revealed that several synthesized compounds 8, 9, 11-13 and 15 as well as indomethacin showed significant antinociceptive effect in comparison with control (P < 0.001) (Table 1). In compounds 7-9, existence of a more lipophilic group on the position 2 of the oxadiazole ring lead to a more potent compound 8. In addition, bioisosteric replacement of oxygen atom in compound 8 with NH to give compound 10, decreased the activity. Replacement of hydrogen atom in 4th position of triazole ring with alkyl or aryl groups, compounds 11-15 caused a noticeable analgesic activity. The most potent analgesic derivative was 15 and it can be concluded that existence of both electron withdrawing and donating substituents on the para position of the phenyl moiety (compounds 13 and 15 ) could enhance the analgesic activity of triazole derivatives.

**Table 1:** Effects of new imidazolyl oxadiazole and triazole derivatives, and Indomethacin in the inhibition of abdominal constrictions induced by acetic acid (%0.6) in Mice

**Compound**	**Constriction no. (mean ± SEM)**^**1**^	**Inhibition (%)**^**2**^	**Relative activity**^**3**^
Vehicle control	59.67 ± 6.8	-	-
Indomethacin	13.50 ± 2.9	77.37	1^***^
7	44.33 ± 8.1	25.70	0.33
8	18.00 ± 3.7	69.83	0.90^***^
9	24.67 ± 5.5	58.65	0.76^***^
10	44.67 ± 2.8	25.14	0.32
11	23.83 ± 4.8	60.06	0.77^***^
12	24.33 ± 3.3	59.22	0.76^***^
13	17.67 ± 2.9	70.39	0.91^***^
14	ND^4^	-	-
15	15.50 ± 3.9	74.02	0.96^***^

^1^All compounds were administered i.p. at the dose of 50 μmol/kg, number of animals in each group n = 6.

^2^% of inhibition obtained by comparison with vehicle control group.

^3^Analgesic activity relative to Indomethacin,^***^P < 0.001 significant as compared to control.

^4^Not determined.

### Anti-inflammatory activity

In vivo anti-inflammatory evaluation of target compounds is summarized in Table 2. Compounds 8-9 and 11-13 were active anti-inflammatory agents (33-43% inhibition) after 5 h in comparison with control and their activity was comparable to indomethacin. As shown in Table 2, there wasn't any significant difference between the activity of compounds 9, 11 and 13 and the most active derivatives were 8 and 12. Similar to analgesic activity results, it can be deduced by comparison of these data that the best substituent on position 2 of oxadiazole ring is a sufur moiety. In contrast to analgesic activity, the introduction of electron donating or withdrawing moieties on the para position of phenyl ring has a deleterious effect on the anti-inflammatory activity.

**Table 2:** Effects of new imidazolyl, oxadiazole and triazole derivatives 50(μmol/kg), and Indomethacin in the Carrageenan-induced rat paw edema

**Compound**	**Time (h)**^**1**^	**Volume variation (μL)**^**2,3**^	**% inhibition**^**4**^
Vehicle control	1	1.53 ± 0.12	-
	2	1.84 ± 0.17	-
	3	2.22 ± 0.24	-
	4	2.44 ± 0.26	-
	5	2.11 ± 0.21	-
Indomethacin	1	1.17 ± 0.01	23.53
	2	1.35 ± 0.01	26.63
	3	1.14 ± 0.02	48.6^**^
	4	1.03 ± 0.02	57.79^***^
	5	0.89 ± 0.01	57.82^***^
8	1	1.60 ± 0.15	-4.60
	2	1.82 ± 0.16	1.10
	3	1.68 ± 0.18	24.32
	4	1.53 ± 0.21	37.30^**^
	5	1.19 ± 0.18	43.60^**^
9	1	1.78 ± 0.14	-16.34
	2	1.90 ± 0.14	-3.26
	3	1.96 ± 0.16	11.71
	4	1.78 ± 0.14	27.05
	5	1.32 ± 0.07	37.44^*^
11	1	1.95 ± 0.04	-27.45
	2	2.11 ± 0.06	-14.67
	3	1.82 ± 0.16	18.02
	4	1.67 ± 0.20	31.56^*^
	5	1.38 ± 0.22	34.60^*^
12	1	1.27 ± 0.04	16.99
	2	1.44 ± 0.06	21.74
	3	1.49 ± 0.04	32.88^*^
	4	1.45 ± 0.07	40.57^*^
	5	1.22 ± 0.08	42.18^*^
13	1	1.19 ± 0.16	22.22
	2	1.70 ± 0.15	7.60
	3	1.55 ± 0.12	30.2^*^
	4	1.66 ± 0.11	31.97^*^
	5	1.41 ± 0.10	33.17^*^
14	1	1.39 ± 0.07	9.15
	2	1.64 ± 0.10	10.87
	3	1.90 ± 0.14	14.41
	4	1.91 ± 0.11	21.72
	5	1.69 ± 0.12	19.90
15	1	1.46 ± 0.08	4.57
	2	1.78 ± 0.08	3.26
	3	1.96 ± 0.10	11.71
	4	2.02 ± 0.26	17.21
	5	1.98 ± 019	6.16

^1^Time after carrageenan injection (0.1 mg/paw), number of animals in each group n = 6.

^2^All compounds were administered i.p, at the dose of 50 μmol/kg.

^3^Volume variation is the difference between the volumes of paw pre and post of Carrageenan injection.

^4^% of inhibition obtained by comparison with vehicle control group, *P < 0.05 , ^**^p < 0.01, ^***^p < 0.001 significant from control.

### Ulcerogenic activity

The compounds 11 and 13 as two active derivatives, in anti-Inflammatory and analgesic tests respectively, were evaluated for their acute ulcerogenic activity. A significant reduction in ulcerogenic activity with the stomach ulceration score between 0.50 ± 0.01 and 0.58 ± 0.08.

was observed in the tested compounds. In fact, not only the standard drug, indomethacin showed a high score of 1.67 ± 0.33, but also none of these two evaluated derivatives induced significant ulceration in comparison to control (Table 3).

**Table 3:** Acute ulcerogenic activity of compounds (11 and 13) in the method of Cioli

**Compound**	**Dose (mg/kg)**^**1**^	**Ulcerogenic activity (Severity index ± SEM)**	**P value**
11	105	0.50 ± 0.01	p > 0.05
13	105	0.58 ± 0.08	p > 0.05
Indomethacin	105	1.67 ± 0.333	P^*^ < 0.001
Control	-	0.00 ± 0.00	-

^1^number of animals in each group n = 6, *p < 0.001 significant from control.

### In silico drug-likeness and toxicity results

In this work, open-source program OPE was used to evaluate the fragment-based drug-likeness of title compounds and comparing them with indomethacin and celecoxib. The overall drug-score which was mentioned earlier combines drug-likeness, clogp, clogs, molecular weight and toxicity risk factors in one single value, where the occurrence frequency of each fragment is determined within the collection of approved drugs and within the supposedly non-drug like chemicals of fluka company. A positive drug-likeness value (0.1-10) states that a molecule contains fragments which are present in commercial drugs. The OPE study revealed that except compound 14 which showed high risk of mutagenic and medium risk of tumorgenic effects, all compounds, indomethacin and celecoxib are supposed to be non-mutagenic, non-tumorgenic, non-irritant with no reproductive effects. The *in silico* drug-relevant properties obtained by OPE are given in Table 4. The potential drug-likeness values of all designed compounds were significantly higher than that of celecoxib but this value for compounds 7 and 9 is negative so their similarity to commercial chemicals is more than traded drugs. Generally, the drug-score values of compounds 7-15 (0.34-0.94) were more than that of celecoxib (0.31) and these scores for compounds 8, 10-13 and 15 (0.68-0.94) were more than indomethacin and for compounds 7 and 9 comparable with indomethacin.

**Table 4:** Drug-likeness of target compounds predicted by Osiris Property Explorer tool in comparison with indomethacin and celecoxib

**Compound**	**Toxicity risk**^**1**^				**cLogP**	**Solubility**	**MW**	**Drug- likeness**	**Drug-score**
	**M**^**2**^	**T**^**3**^	**I**^**4**^	**R**^**5**^					
7	-	-	-	-	0.06	-2.57	165	-1.88	0.54
8	-	-	-	-	0.92	-2.2	182	0.87	0.81
9	-	-	-	-	0.25	-2.12	166	-1.75	0.55
10	-	-	-	-	0.75	-2.06	181	1.39	0.86
11	-	-	-	-	1.04	-1.7	195	2.39	0.94
12	-	-	-	-	2.29	-3.55	257	2.46	0.82
13	-	-	-	-	2.61	-3.89	271	0.76	0.68
14	**+**	**±**	-	-	2.19	-3.56	287	0.7	0.34
15	-	-	-	-	2.35	-3.86	275	0.7	0.69
Indomethacin	-	-	-	-	3.83	-5.4	357	7.59	0.57
Celecoxib	-	-	-	-	2.27	-5.4	365	-9.50	0.31

^1^Ranked according to: (-) no bad effect, (**±**) medium bad effect, (+) bad effect; ^2^ M, Mutagenic effect; ^3^ T, Tumorgenic effect; ^4^I, Irritating effect; ^5^R, Reproductive effect.

## Conclusion

A series of new methyl-imidazolyl-1,3,4-oxadiazoles and 1,2,4-triazoles were designed and synthesized. The *in vivo* analgesic, anti-inflammatory and ulcerogenic evaluations, revealed that most of them were active in both tests without any ulcerogenic potential in comparison to control. The results revealed that both of oxadiazole and triazole scaffolds can be determined as pharmacophores. However, compounds 11 and 12 showed the highest values of drug-likeness and drug-score and acceptable pharmacological activity in both tests, so they could be selected as lead compounds for further modifications.

### Statistics

Statistics were performed with one-way analysis of variance (ANOVA), which was followed by Tukey multi-comparison test. All data are presented as mean ± SEM, p < 0.05 was considered to be significant.

## Competing interests

The authors declare that they have no competing interests.

## Authors’ contributions

AA: Design of target compounds, supervision of the synthetic part and manuscript preparation. ZM: Supervision of pharmacological part. MT: Synthesis of target compounds. MJA: Performed the pharmacological tests. AS: Collaboration in identifying of the structures of target compounds. All authors read and approved the final manuscript.
